# Influence of Time Pressure on Successive Visual Searches

**DOI:** 10.3390/jemr18040031

**Published:** 2025-07-17

**Authors:** Alejandro J. Cambronero-Delgadillo, Christof Körner, Iain D. Gilchrist, Margit Höfler

**Affiliations:** 1 Department of Psychology, University of Graz, 8010 Graz, Austria; christof.koerner@uni-graz.at (C.K.); ma.hoefler@uni-graz.at (M.H.); 2 School of Psychological Science, University of Bristol, Bristol BS8 1QU, UK; i.d.gilchrist@bristol.ac.uk; 3 Department for Dementia Research and Care Science, University for Continuing Education Krems, 3500 Krems, Austria

**Keywords:** eye movements, eye tracking, time pressure, visual search, interruption

## Abstract

In the current eye-tracking experiment, we explored the effects of time pressure on visual search performance and oculomotor behavior. Participants performed two consecutive time-pressured searches for a T-shaped target among L-shaped distractors in two separate displays of fifteen items, with the option to self-interrupt the first search (Search 1) to proceed to the second (Search 2). Our results showed that participants maintained high search accuracy during Search 1 across all conditions, but performance noticeably declined during Search 2 with increasing time pressure. Time pressure also led to decreased numbers of fixations and faster response times overall. When both targets where acquired, fixation durations were longer in Search 2 than in Search 1, while saccade amplitudes were shorter in Search 2. Our findings suggest that time pressure leads to the first target being prioritized when targets possess equal value, emphasizing the challenges of optimizing performance in time-sensitive tasks.

## 1. Introduction

Imagine you are in a supermarket, searching for a bottle of wine for a dinner party. As you stand before the wine selection, a notification reminds you to pick up a bouquet from a florist that is closing soon. With the dinner party’s start-time approaching, you must make a critical decision: do you fully commit to finding the best wine, risking missing the florist? Do you quickly search for the wine, planning to leave once you have spent enough time, then proceed to the florist? Or do you immediately prioritize getting the bouquet and abandon the wine search?

Each option leads to different outcomes. Spending all your time on the wine may help you find the desired bottle, but you may not reach the florist in time. A short search for the wine followed by a visit to the florist tries to handle both tasks, but it might mean you do not complete either task fully. Prioritizing the bouquet ensures you will obtain the flowers, but you will not have selected the wine. In the end, each choice requires balancing the importance of securing the wine with the need to have the flowers for the dinner party.

Visual search—the process of locating a target amidst distractors [1]—is a common activity in our daily lives. Examples include searching for a particular letter on a keyboard, spotting a familiar face in a crowd, or trying to find a bottle of wine in the supermarket. In experimental studies, visual search is commonly examined using tasks such as finding a target letter among a group of others [2,3,4], searching for a specific word on a list [5,6]), or identifying an image among a collection of images [7,8,9]. Decades of research have established that visual search is significantly influenced by factors such as color, motion, orientation, and size; for a review, see [10,11]. For example targets with a unique feature are identified more rapidly than those that share features with the distractors [12,13,14,15]. This search advantage for unique features was early explained by Texton theory [16], feature integration theory [17] and guided search theory [18]. That is, Texton theory suggests that basic visual features (“textons”, such as specific orientations or colors) are detected early in visual processing, allowing for rapid and automatic identification. Following this initial detection, the visual system engages slower, serial attentive processes in order to analyze the spatial relationships among the features within a limited focus of attention, thus enabling detailed form recognition [16,19]. In a similar approach, Treisman’s feature integration theory [17] proposes that basic features such as color and shape are processed in parallel and preattentively. That is, a target with a unique feature (such as color) “pops out” while targets sharing features with distractors require a serial search and attention, leading to slower identification. Similarly, Guided Search theory [18] proposes that the initial, parallel processing of basic features generates an activation map that guides the subsequent deployment of attention toward the likeliest target locations.

In addition to the well-established factors such as color, motion, orientation, and size, time pressure has been proposed to modulate the search process [20]. For instance, Yu et al. [21] found that participants searching for a target in a display of slow- or fast-moving items responded faster under time pressure, but only in the slow-moving condition. Time pressure has also been shown to improve response accuracy in computer-assisted searches [22] and to speed up responses without compromising accuracy [23]. Furthermore, McCarley [24] conducted a simulated baggage-screening task, instructing participants to prioritize either speed or accuracy. Participants who focused on speed made fewer and shorter fixations, coupled with longer saccades, which led to more frequent target misses—despite their ability to recognize targets when fixated. Another example of the impact of time pressure can be seen in the study by Van Herpen and Trijp [25], where participants were asked to select a cereal based on nutritional labels under varying levels of time pressure. The results revealed that under high time pressure, participants were less likely to fixate on the labels, spent less time examining them, and processed less overall information compared to low time-pressure conditions. Similarly, Rieger and Manzey [26] examined participants’ performance in a simulated X-ray screening task, where they searched for a target letter under low and high time pressure. Their findings demonstrated that high time pressure led to decreased target detection performance and reduced exploration of the search area compared to low time pressure.

Another critical factor that influences search behavior is the interruption of an ongoing search; for a review, see [27]. Research has shown that participants can quickly resume and complete a search task after a momentary interruption when the same display reappears. This phenomenon, known as the “rapid resumption” effect in visual search, was first observed by Lleras et al. [28] and has been systematically replicated and expanded upon since [29,30]. The effect is thought to occur because participants form an initial hypothesis during their first exposure to the search display, which they then test during subsequent exposures. Even when the search display is temporarily hidden, participants appear to continue the search process, as evidenced by their ongoing saccade planning during the interruption phase [31]. Furthermore, studies have demonstrated that participants can inhibit refixation on previously attended items (i.e., inhibition of return) despite interruptions during the search task [32]. Overall, these findings suggest that participants use the information gathered before an interruption to enhance their search efficiency when the display reappears.

A common feature of these experiments is that participants were shown either the original display or a slightly modified version after the interruption. This setup allowed them to complete their initial search before transitioning to the next search. Moreover, the interruption manipulations were exogenous, meaning they were imposed on participants without their control. Finally, the primary focus of these studies was on the impact of interruptions on search resumption—specifically, the time between resuming the search after the interruption and the participant’s manual response to the target. However, in real-life visual searches, we often need to interrupt an ongoing search to initiate a new one due to changing priorities. In such situations, we must quickly assess whether it is more beneficial to abandon the current search and start a new task, considering factors such as the urgency and relevance of the new task, as well as the feasibility of switching. For example, returning to the earlier dinner party scenario, if you are running late and cannot find the bottle of wine, you might decide to stop searching for the wine and proceed to the florist instead. While voluntarily switching searches due to time constraints is a common occurrence in daily life, there is limited research on this specific type of search behavior. As a result, the effects of time pressure and incomplete searches on subsequent search activities remain largely unexplored.

In the current eye-tracking experiment, we investigated the effect of varying levels of time pressure on visual search by having participants perform two consecutive, time-limited searches for a target in two separate displays of fifteen items. When searching under time pressure, participants had the choice of self-interrupting Search 1 to move on to Search 2. In line with previous findings, we anticipate a decrease in response accuracy in both searches as time pressure increases [33]. It remains an open question how participants will allocate their time across the two searches under different time constraints. If they prioritize completing Search 1 over Search 2 (a likely scenario given the high chance of running out of time), they might also spend more time on Search 1 as time pressure increases, leading to more items fixated in Search 1 than in Search 2. We expected that fixation duration would not differ between Search 1 and Search 2 but might shorten with increased time pressure [34]. Additionally, we believe that saccades would become overall smaller with increasing time pressure [35]; however, it remains an open question whether saccade lengths also differ between Search 1 and Search 2. Additionally, while there is evidence demonstrating participants’ ability to quickly and efficiently resume externally interrupted search tasks [29], we were also interested in how and to what extent participants employ a self-interruption strategy during Search 1 to proceed to the next search.

## 2. Materials and Methods

### 2.1. Participants

In total, we tested 24 participants (22 female, 2 male, *M* = 23.5 years, *SD* = 3.32). In order to estimate the sample size using an ANOVA (repeated measures, within factors), we utilized G*Power 3.7.9.7 [36]. Using an α of 0.05, a power of 0.8, and an effect size of *f* = 0.31, we determined that a total of 24 participants were needed for the study. All participants reported normal or corrected-to-normal vision (contact lenses). Participants either received monetary compensation (10 Euro/h) or course credit for their participation. Prior to testing, written informed consent was obtained from all participants. The study was approved by the ethics committee of the University of Graz.

### 2.2. Apparatus

A tower-mounted EyeLink 1000 Plus eye tracker (SR Research, Mississauga, ON, Canada) was used to track the eye movements of the participants. To discourage head movements, a chin and forehead rest were utilized. The viewing distance to the stimulus screen was set to 63 cm. Eye movements were recorded at a sampling rate of 1000 Hz. The velocity threshold for saccade detection was set to 35°/s, and the acceleration threshold to 9500°/s^2^. At the start of each block, a standard 9-point binocular calibration took place. The eye that produced the better spatial resolution (average error < 0.3°) was chosen for data collection. The experimenter controlled the procedure with a computer attached to the eye tracker via an Ethernet connection. Search displays were presented on a 24” monitor with a refresh rate of 144 Hz and a resolution of 1920 × 1080 pixels. Manual responses were collected with a Microsoft SideWinder^®^ gamepad (Microsoft Corporation, Redmond, WA, USA).

### 2.3. Design

This study was preregistered on the Open Science Framework (OSF; https://osf.io/nhztw) prior to data collection. We conducted the experiment in a two-factorial design, with four levels of time pressure (baseline, low time pressure, medium time pressure, and high time pressure) and two consecutive searches in two different displays within one trial (Search 1 and Search 2). Note that including Search as a second factor reflects a deviation from the originally planned design. Participants were asked to search for a T-shaped target among L-shaped distractors and to report its rotation (clockwise/anticlockwise). The target was always present. Time limits for completing both searches were individually tailored based on participants’ baseline performance. In the low time-pressure condition, the time limit (across the two searches) was set to their mean baseline time. For the medium and high time-pressure conditions, the time limits were reduced by half a standard deviation and one standard deviation from their mean, respectively.

### 2.4. Materials

Each search display consisted of 15 items: 14 light gray “L” letters and 1 light gray “T” letter (Arial font, bold; RGB: 130, 130, 130), the latter serving as a target in all searches. Each of the “L” distractors as well as the target “T” were presented tilted to the right (at 45° from their upright position) or to the left (at 315° from their upright position) randomly in each search. Each letter subtended 0.32° and was encircled in a 0.18° thick circle with an outer diameter of 0.9°. The circle carried a twofold purpose; it outlined a defined object to aid saccade planning and helped to reduce parafoveal preprocessing of the stimuli [37]. The letters were randomly placed on the intersection of an invisible 6 × 6 grid, with each grid cell having a size of 3.6°. Each letter position was randomly jittered vertically and horizontally in each trial by a ± 0.23° deviation from the intersection. The stimuli were presented on a black background.

### 2.5. Procedure

**Figure 1:**
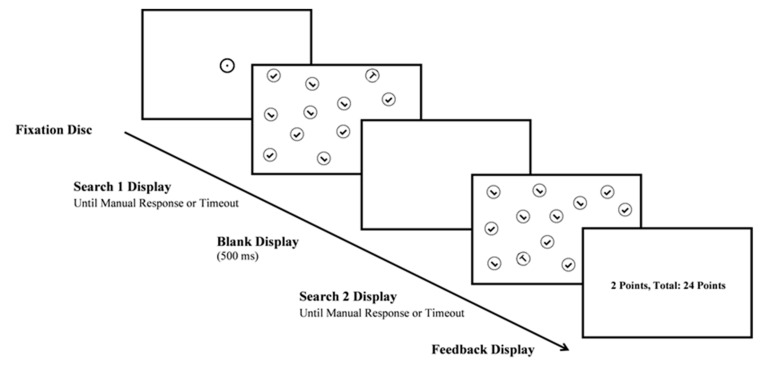
Sequence of events in an experimental trial. After a timeout or manual response during Search 2, score feedback was displayed. Stimuli are not to scale.

Participants were seated facing the display screen in a soundproof, darkened cabin. At the start of each trial, a fixation disc was displayed at the center of the screen. Participants were asked to fixate on the disc to correct drift. Each trial was initiated once the experimenter manually accepted the fixation. The participant’s task was to search for the T-shaped target in the display and report its orientation via a button press. If the target was rotated clockwise, the participant was instructed to press the right trigger of the controller with the right index finger. If the target was rotated anticlockwise, the participant was instructed to press the left trigger with the left index finger. Each target correctly identified awarded participants one point, with a maximum of two points per trial. If the participant decided to interrupt the Search 1, they pressed the “A” button with the right thumb. With this button press, the first display was cleared, and a blank display appeared onscreen for 500 ms, before the Search 2 display was presented. Then, participants searched again for the T among the Ls and reported its orientation. After Search 2, the display was cleared and a feedback display with the number of points for the current trial and the total points for the block was displayed for 2000 milliseconds before a new trial began (see Figure 1).

In the baseline block, participants were required to search for both targets as fast and as accurately as possible. This block was conducted without any time restriction, and we calculated the average response time and the standard deviation for each participant in this block. In Blocks 2–4, participants were informed about a time constraint, specifically that they would have “limited time for the two searches” before the first time-pressure block and “slightly more (less) time than in the last block” to complete the two searches that comprised a trial. Nevertheless, they were asked to complete both searches to the best of their abilities and to consider the time allotted to each search. Participants completed four blocks with 100 trials each, for a total of 400 trials per participant. The order of presentation of each time pressure condition (low, medium, and high) was counterbalanced across participants. Each session lasted approximately 90 min.

## 3. Results

We collected 9600 trials (400 trials × 24 participants). A total of 409 trials were lost due to technical errors. Due to the low prevalence of the use of the self-interruption mechanism (1%, 2%, and 2% in the low, medium, and high time-pressure conditions, respectively), we opted to exclude trials in which it was used. To ensure participants understood and correctly completed the task, we checked the incorrect response rates (i.e., trials in which participants incorrectly reported the target orientation) in the baseline block. The incorrect response rate was 1%, indicating proper task comprehension and completion. Furthermore, in the baseline block, it took participants on average 6118 ms (*SD* = 623) to complete both searches, with individual times ranging from 5094 ms to 7212 ms. Participants allocated 2918 ms (*SD* = 336) on average to Search 1 (range: 2296–3661 ms) and almost the same amount of time to Search 2 (*M* = 3190 ms; *SD* = 340; range 2638–3813). Furthermore, participants became progressively faster with repeated trials in the baseline condition, *r* (100) = −0.217, *p* = 0.030 (Spearman’s correlation).

Although the individual time pressure levels were determined based on the average of 100 trials in the baseline block, which should have ensured a certain stability of this average, the increased time pressure levels could nevertheless have been differently demanding for participants. Thus, for better comparability, we decided to normalize the response time data to test how participants allocated the time to the individual searches in a trial. For the following sections, we are going to discuss solved trials, defined as those where participants correctly identified the targets in both Search 1 and Search 2 within the allotted time. For data analysis, we used JAMOVI 2.3.28 [38]; the alpha level was set to 0.05.

### 3.1. Search Accuracy

**Figure 2:**
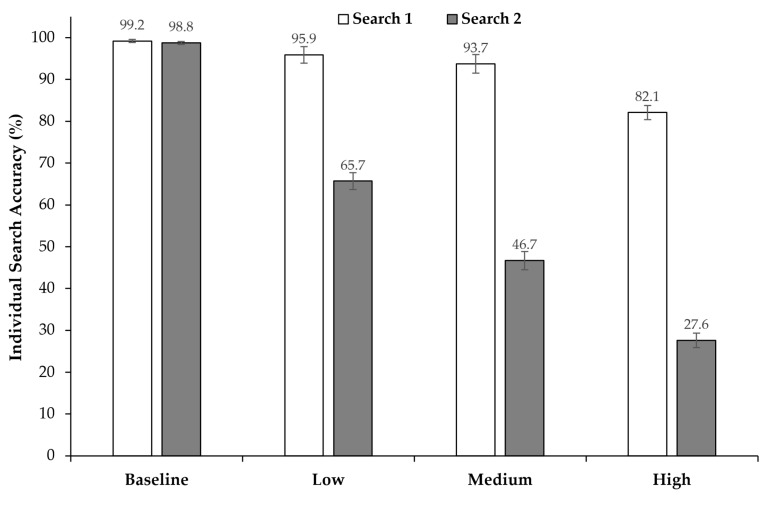
Average search accuracy for time pressure conditions and search (Search 1 vs. Search 2). Error bars represent 95% confidence intervals [39,40].

Overall, the percentage of solved trials decreased as time pressure increased (see Figure 2). When comparing individual search accuracy (i.e., Search 1 vs. Search 2), we observed that accuracy in Search 1 remained relatively consistent across time pressure conditions, with only a small decline under high time pressure. In contrast, accuracy in Search 2 showed a steep decline as time pressure increased. To determine whether solved rates differed significantly between time pressure conditions and between the first and second search, we performed a two-way repeated measures ANOVA with time pressure (baseline, low, medium, high) and search (Search 1, Search 2) as factors. Time pressure had a significant effect on accuracy, *F*(3, 69) = 458; *p* < 0.001, η2p = 0.952. Additionally, there was also a main effect of search, *F*(1, 23) = 1938; *p* < 0.001, η2p = 0.988. An interaction effect was also found, *F*(3, 69) = 301; *p* < 0.001, η2p = 0.929, indicating that Search 2 was, with increasing time pressure, significantly more impacted than Search 1.

### 3.2. Number of Fixations and Response Times

**Figure 3:**
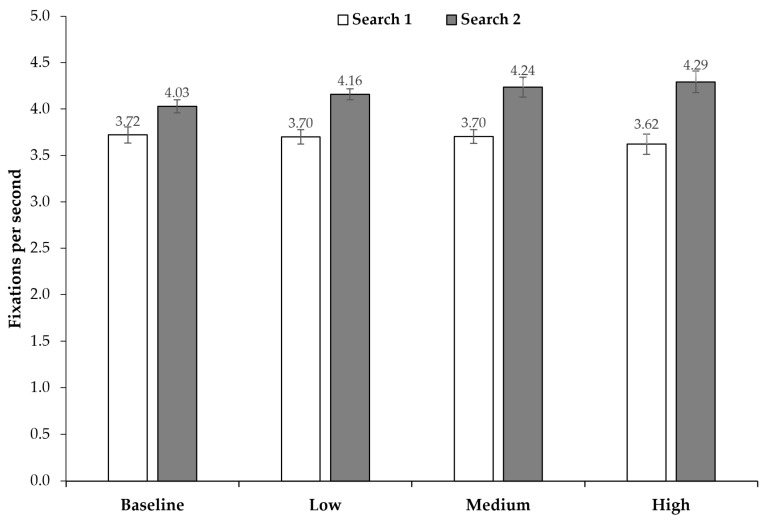
Average number of fixations per second across time pressure conditions and search (Search 1 vs. Search 2). Error bars represent 95% confidence intervals [39,40].

We first analyzed the number of fixations per second and response times (for solved trials only) during searches under different levels of time pressure. Overall, the number of fixations per second seemed to be consistently higher in Search 2 compared to Search 1—regardless of time pressure conditions (Figure 3).

**Figure 4:**
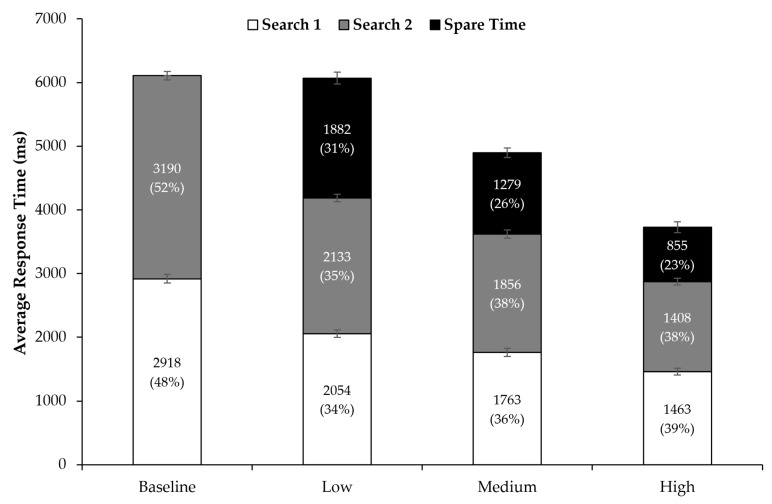
Mean response times and percentage of time use across time pressure conditions and search (Search 1, Search 2, and Spare Time). Error bars represent 95% confidence intervals [39,40].

Furthermore, with regard to the manual response times, it seems that, descriptively, search times decreased with increasing time pressure while Search 2 did not seem to last longer than Search 1 in any of the time pressure conditions (see Figure 4). Spearman correlations revealed that search times did not become faster across a block in the time pressure conditions (all ps > 0.05), except for a weak effect in Search 2 under medium time pressure, r(100) = 0.200, *p* = 0.046. However, as the time pressure levels were individualized, it is difficult to compare the response times directly across the time pressure conditions. Therefore, we decided to normalize the search times by dividing them by the individual maximum time for statistical analysis. This resulted in percentage scores for the relative time taken in each search (and the time left during the trial; see Figure 4). In the baseline condition, participants allocated on average 48% (SD = 1.3) of their time to Search 1 and 52% (SD = 2.0) to Search 2. Under low time pressure, the allocation shifted to 34% (SD = 2.2) for Search 1 and 35% (SD = 2.5) for Search 2. With medium time pressure, participants spent 36% (SD = 2.9) of their time on Search 1 and 38% (SD = 3.0) on Search 2. Finally, under high time pressure, the time allocation was 39% (SD = 2.7) for Search 1 and 38% (SD = 3.2) for Search 2.

### 3.3. Fixation Durations

**Figure 5:**
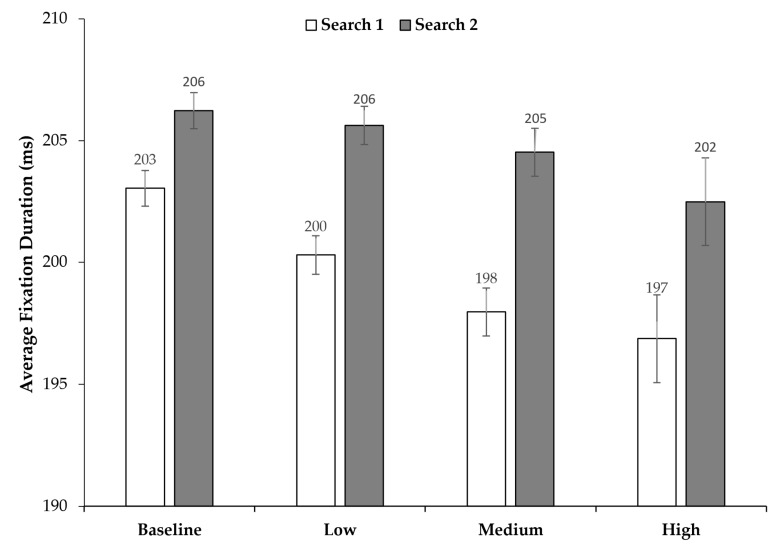
Average fixation duration across time pressure conditions and search (Search 1 vs. Search 2). Error bars represent 95% confidence intervals [39,40].

For analysis of the fixation durations, we did not consider the first fixation in a trial and also excluded fixations on the target. Average fixation durations for the two searches, depending on the time pressure conditions, can be found in Figure 5. Descriptively, fixation durations seem to be slightly longer in Search 2 than in Search 1. To examine the effect of time pressure and search order on fixation duration, we performed a 2 × 4 repeated measures ANOVA with time pressure (baseline, low time pressure, medium time pressure, high time pressure) and search (Search 1, Search 2) as factors. Fixation durations in Search 2 were significantly longer than in Search 1, *F*(1, 23) = 12.180; *p* = 0.002, η2p = 0.346. There was no main effect of time pressure, *F*(3, 69) = 1.912; *p* = 0.136. Also, no interaction was found, *F*(3, 69) = 0.480; *p* = 0.697.

### 3.4. Saccade Amplitudes

**Figure 6:**
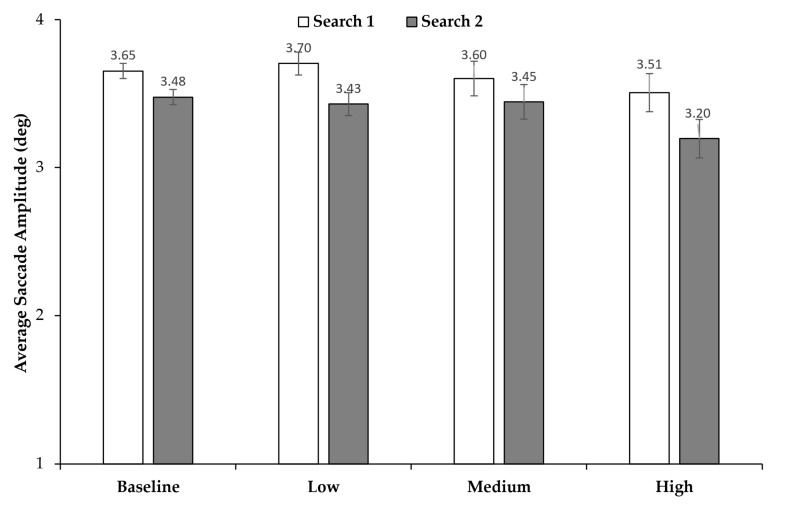
Average saccade amplitude across time pressure conditions and search (Search 1 vs. Search 2). Error bars represent 95% confidence intervals [39,40].

Overall, the amplitude of the saccades seemed to remain relatively consistent across time pressure conditions, with a slight decrease in Search 2 compared to Search 1 (see Figure 6). In order to assess whether these differences were significant, we conducted a two-way repeated measures ANOVA with time pressure (baseline, low time pressure, medium time pressure, high time pressure) and search (Search 1, Search 2) as factors: participants performed longer saccades in Search 1 compared to Search 2, *F*(3, 69) = 19.06; *p* < 0.001, η2p = 0.453. There was also a main effect of time pressure *F*(3, 69) = 9.26; *p* < 0.001, η2p = 0.287. No interaction was found, *F*(3, 69) = 1.85; *p* = 0.146. Bonferroni-corrected post-tests revealed a significance difference between the baseline and the high time-pressure conditions (*p* < 0.001), the low and high time-pressure conditions (*p* = 0.003), and the medium and high time-pressure conditions (*p* = 0.04). No other differences were significant (all *ps* = 1.000). Together, these findings suggests that saccades were overall shorter in Search 2 than in Search 1 and that only high time pressure led to shorter saccades.

There is evidence that saccade amplitude, peak velocity, and saccade duration are interrelated, with peak velocity and duration increasing linearly with amplitude—a relationship known as the main sequence [41]. The linear amplitude–velocity–duration relationship holds consistently for saccades below 30° [42,43]. However, at the same time, there seems to be a high inter- and intraindividual variability in this relationship between peak velocity and amplitude [44,45], suggesting that the oculomotor system incorporates flexible modulation of saccadic dynamics based on task demands [46]. In their experiment, Di Stasi et al. [47] analyzed the three parameters of the main sequence separately in order to test possible effects of different mental workload levels during a driving situation and showed that only peak velocity was affected by mental workload—but that there was no interaction between task complexity and saccade length with regard to peak velocity.

**Figure 7:**
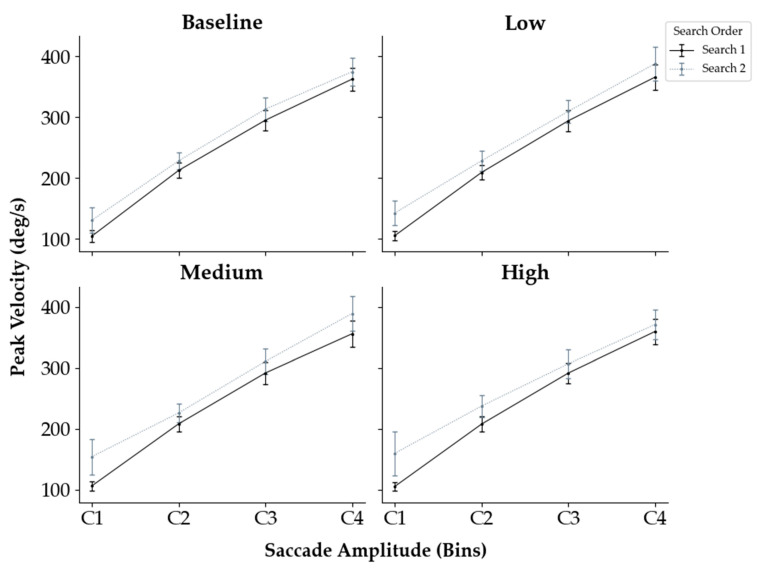
Average peak velocity as a function of binned saccade amplitudes across time pressure conditions and search (Search 1 vs. Search 2). Error bars indicate 95% confidence interval (CI).

We therefore explored whether time pressure and search conditions differentially affected peak velocity across saccade amplitudes (Figure 7). We categorized amplitudes into four bins (0.01–2°, 2.001–4°, 4.001–6°, and 6.001–10°) and conducted a 4 (time pressure) × 2 (search) × 4 (amplitude bin) repeated-measures ANOVA on the median peak velocity. The analysis revealed a significant three-way interaction, *F*(9, 207) = 2.243, *p* = 0.021, ηp2 = 0.089. Significant two-way interactions were also found between time pressure and amplitude, *F*(9, 207) = 6.049, *p* < 0.001, ηp2 = 0.208, and between search and amplitude, *F*(3, 69) = 13.974, *p* < 0.001, ηp2 = 0.378. Additionally, there was a main effect of amplitude, *F*(3, 69) = 870.907, *p* < 0.001, ηp2 = 0.974. No main effects were found for time pressure (*p* = 0.800) or search (*p* = 0.068). The interaction between time pressure and search was also not significant (*p* = 0.678). Bonferroni corrected post hoc comparisons indicated that peak velocity increased significantly with each successive amplitude category (*p* < 0.001 for all comparisons). Regarding the interaction, significant differences between searches and conditions were only found for the shortest amplitudes (C1) such that, in the baseline condition, peak velocities in Search 2 were higher than in Search 1 (*p* = 0.006) and smaller than in Search 2 high-time pressure searches (*p* = 0.009).

## 4. Discussion

In the current experiment, we investigated the impact of time pressure on visual search performance and oculomotor behavior. Participants completed two consecutive “T among Ls” searches under varying time pressure conditions. We observed that search performance declined progressively with increased time pressure, with a more significant drop in performance during the second search compared to the first. Additionally, time pressure also affected response times, as participants not only responded faster with increasing time pressure but also used more of the available time to complete the searches. Furthermore, the analysis of the eye movements revealed that participants fixated more items and fixation durations were longer in Search 2 compared to Search 1. At the same time, saccade amplitudes became shorter in Search 2 and with increasing time pressure.

Consistent with our initial hypothesis, time pressure exerted a negative impact on search accuracy, with the detrimental effect intensifying as the level of time pressure increased. This finding aligns with the existing literature, which has consistently demonstrated that time pressure adversely affects search accuracy. For example, Fan et al. [20] found that time pressure significantly impaired search accuracy in a simulated visual detection task. Additionally, Rieger et al. [33] reported that time pressure reduces thoroughness in visual search, leading participants to explore a smaller area and to have diminished target detection performance. However, in those studies, typically, the effect of time pressure on a single visual search was tested. Here, we can add to these findings that this overall detrimental effect did not apply to two consecutive searches to the same extent. That is, in all time pressure conditions, Search 1 demonstrated quite high accuracy, with more than 90% correct under low and medium time pressure and still over 80% under high time pressure, while in Search 2, accuracy decreased from ca. 67% to only 28%. Together, this suggests that participants prioritized Search 1 over Search 2.

This observed prioritization of Search 1, leading to a noticeable decline in Search 2 accuracy under increasing time pressure, bears conceptual similarities to the ‘attentional blink’ (AB) phenomenon [48]. The AB describes a deficit in reporting a second target when it appears in close temporal succession after a first target, an effect attributed to limitations where the processing and consolidation of the first target consumes attentional resources [49]. While the AB is traditionally investigated using Rapid Serial Visual Presentation (RSVP) streams, recent evidence demonstrates that an analogous, “self-induced” attentional blink [50] can occur in self-paced, spatial visual searches that more closely resemble our paradigm. Adamo et al. [50] found that even when participants controlled their own scan paths, accuracy for a second target in the same display was significantly impaired when it was fixated within a few hundred milliseconds of the first. In our study, the completion of Search 1 could be considered the resource-demanding first target event. This raises the possibility that the decline in Search 2 accuracy is related to an AB-like period of impaired processing—at least under time pressure. The concept of a “self-induced” processing deficit, therefore, offers a potential cognitive explanation for why participants prioritized Search 1 in while time constrained. However, because our paradigm involved two distinct search tasks, this interpretation should be approached with caution, and further research is necessary to more systematically investigate this possible effect of time pressure across two searches.

In addition, descriptively, the search times decreased, as expected, with higher time pressure. Interestingly, participants did not seem to be able to adapt to the time pressure level within a block. As mentioned above, participants were told they would have “limited time for the two searches” before the first time-pressure block and “slightly more (less) time than in the last block” before the next; however, the terms ‘limited,’ ‘less,’ and ‘more’ were not explained in detail. In the baseline condition, the absence of time constraints enabled participants to refine their search strategies through deliberate practice across the trials in the block. However, under pressure, we observed no such effect as the limited time available may have constrained their ability to refine strategies. Indeed, the time allocation analysis revealed that greater time pressure resulted in increased time allocated for searches and consequently less spare time. Still, this contrasts with previous research that showed that participants typically improve with practice [51].

Despite the effect of time pressure on the search times, Search 1 and Search 2 seemed to last equally long in all conditions—while slightly more fixations per second were made during Search 2 compared to Search 1. As stated above, the comparison of response times across time pressure conditions should be made with care because we individualized time pressure for each participant. Nevertheless, in alignment with the raw response times and contrary to our expectations, search times were, at least at first glance, allocated quite equally across the two searches and none of the search seemed to be prioritized over the other after normalizing them. However, in all these trials included in the analysis, both searches were completed successfully. Thus, it is reasonable to assume that the longer the first search took, the higher the probability was that the second search was not solved as time ran out. Indeed, exploratory re-analysis of the data showed that less time was on average allocated to Search 1 when Search 2 could be correctly solved than when not (*p* < 0.001). If Search 2 was solved correctly, only a minor number of Search 1 searches were incorrectly completed (less than 1%). In other words, to maximize the chances of finding both targets, it seems best to equally balance the time spent on each search, though success is not certain. However, it cannot be assumed that, under time pressure, participants can finely control the time allocation for both searches. In future studies, it could be investigated to what extent external feedback (e.g., time indication on the display) could improve search performance [52].

Still, the question remains why time allocation was almost the same for Search 1 and Search 2 while slightly more fixations per second were made during Search 2 compared to Search 1. Also, we did observe a slight (on average about 5 ms) but statistically significant increase in fixation duration in Search 2 compared to Search 1, which should also have extended the search time in Search 2. Contrary to the findings of earlier studies [34], we did not observe a change in fixation duration as time pressure increased. It could be that, as the targets required the same amount of processing as distractors for identification, maintaining a consistent fixation duration regardless of the increasing time pressure was necessary to complete the task. The inconsistency between time allocation, number of fixations per second, and search durations across searches might be explained by the fact that, overall, the number of fixations is a greater determinant of the search time than fixation durations [53] and that the amplitudes of the saccades in Search 2 were significantly smaller than the amplitudes in Search 1. This may have led to a trade-off between the number of fixations and the response time, such that more but shorter saccades were made. This further suggests that, due to the limited remaining time in Search 2, participants may have reduced their effort in actively locating the Search 2 target compared to Search 1 such that they preferred searching through items adjacent to their current fixation (see also [33]). However, we did find this pattern regardless of time pressure, i.e., also in the baseline condition. Nevertheless, it could be interesting to investigate in further studies whether the participants actually initiate an active search (i.e., with an expectation of finding the target) when aware that there is little time left for the next search or if they apply a kind of “just-look-and-see” strategy (similar to a “sit-and-wait” strategy in dynamic searches [54]) for the items nearby the current fixation—which, in turn, could also enforce an attentional blink-like effect (see above).

While there was an expected increase in the peak velocity with increasing saccade amplitude, it seems that time pressure did not affect peak velocity. This is in contrast to our assumption and the findings of Di Stasi et al. [47], who showed that differences in mental workload levels during a driving situation modulated peak velocity. However, it could be that time pressure differs from mental workload by acting primarily as a modulator of task pacing rather than inducing the same type of cognitive complexity [23]. Therefore, future research should aim to systematically disentangle the specific effects of task pacing from those of cognitive complexity on oculomotor control.

Participants rarely opted for interrupting Search 1. Considering our findings, there could be various explanations as to why participants did not employ the self-interruption option. First, participants were knowingly searching in displays with a single and present target, where search termination is typically straightforward—that is, once the target is acquired [55]. Second, not only were the displays of equitable search difficulty (i.e., equal set size and same target and distractors), but the expected value of the first and the second target was the same. Therefore, when participants had already searched for some items and were closer to finding the target in Search 1, they perhaps lacked the motivation to interrupt an ongoing search with the same or potentially higher perceived value in order to pursue a new and possibly more uncertain second search. This aligns with findings in voluntary task-switching paradigms where the perceived cost of switching, in the absence of a clear motivational incentive, can lead to task perseverance [56]. As mentioned, the preferred search strategy appears to be to spend as much time as necessary thoroughly searching the first display until the target was found. This decision process could be influenced by a motivational trade-off: the perceived cost of disengaging from the current, seemingly manageable Search 1 may have implicitly outweighed the uncertain benefit or additional effort of switching to Search 2. In a study by Wolfe et al. [57], participants searched for multiple instances of different target types and were free to switch between them. Their results showed that participants tended to focus on collecting targets of the same type, driven by a significant “switch cost”; changing from one target type to another was inefficient, resulting in substantially longer response times. Although the foraging tasks in that study did not involve external time pressure like ours, the concept of a high intrinsic cost for switching provides a strong rationale for our participants’ behavior. The time pressure in our experiment would likely amplify the motivation to avoid an already costly cognitive switch, reinforcing the strategy to complete Search 1. Thus, only after acquiring the first target, and if there was still time remaining, did participants move on to the second display with the aim of finding the second target.

The consistent value of the targets across searches might have also played a role in the observed search behavior. While thoroughly searching the first display until target acquisition seems to be the default strategy under these conditions, it remains to be explored whether this strategy holds true for other search scenarios. For example, in an unbalanced reward scheme consecutive search, where each display’s target holds a different value, participants may exhibit a preference for searching in the display that offers a higher expected reward [58]. Additionally, scenarios where the reward associated with finding a target changes dynamically [59] can also be explored, with the aim of potentially observing how participants’ interruption behavior or search persistence is modulated based on shifting priorities [55]. Beyond target value, differences in the set size between searches could also influence strategic choices, as participants may display a preference for the display with a smaller set size, as it reduces the cognitive load and search effort required [17].

Further research is necessary to investigate situations in which timely self-interruption might constitute the optimal course of action. For example, in real-life scenarios where rapid target identification and subsequent dismissal or pursuit across multiple, successive searches is key, such as in airport security [24] or military surveillance [22], strategic self-interruption could be crucial for optimizing overall performance. Finally, future studies could also examine the role of individual differences [60] as well as expertise [61] in search performance, considering how varying cognitive styles or acquired skills might influence the propensity and effectiveness of self-interruption strategies under time pressure.

## 5. Conclusions

In the current experiment, we examined how varying levels of time pressure affect visual search performance. Participants conducted two consecutive, time-limited searches for a target within two separate displays, with the option to self-interrupt the first search to transition to the second. Time constraints hindered their ability to adapt search strategies, reducing flexibility and limiting potential performance improvements. Our results show how pressure-driven prioritization of speed over adaptability might impact decision-making, emphasizing the challenges of optimizing performance in time-sensitive tasks. Future research could explore how task complexity (i.e., set size) and varying incentives (i.e., different or dynamic target values) influence strategy selection under similar constraints. Additionally, future studies could investigate situations where timely self-interruption is critical, as well as the role of individual differences and expertise in search performance, considering how cognitive style or skill influence self-interruption strategies.

## Data Availability

Dataset available on request from the authors.

## References

[1] Eckstein M.P. (2011). Visual Search: A Retrospective. J. Vis..

[2] Höfler M., Gilchrist I.D., Körner C. (2014). Searching the Same Display Twice: Properties of Short-Term Memory in Repeated Search. Atten. Percept. Psychophys..

[3] Horowitz T.S., Wolfe J.M. (1998). Visual Search Has No Memory. Nature.

[4] Körner C., Gilchrist I.D. (2007). Finding a New Target in an Old Display: Evidence for a Memory Recency Effect in Visual Search. Psychon. Bull. Rev..

[5] Lawrence D.H. (1971). Two Studies of Visual Search for Word Targets with Controlled Rates of Presentation*. Percept. Psychophys..

[6] Radhakrishnan A., Balakrishnan M., Behera S., Raghunandhan R. (2022). Role of Reading Medium and Audio Distractors on Visual Search. J. Optom..

[7] Höfler M., Hübel B. (2018). Missing Targets in Multiple-Target Search. Psychology Applications & Developments.

[8] Stankov A.D., Touryan J., Gordon S., Ries A.J., Ki J., Parra L.C. (2021). During Natural Viewing, Neural Processing of Visual Targets Continues throughout Saccades. J. Vis..

[9] Yang H., Zelinsky G.J. (2009). Visual Search Is Guided to Categorically-Defined Targets. Vis. Res..

[10] Wolfe J. (2020). Visual Search: How Do We Find What We Are Looking For?. Annu. Rev. Vis. Sci..

[11] Wolfe J.M., Horowitz T.S. (2017). Five Factors That Guide Attention in Visual Search. Nat. Hum. Behav..

[12] Bravo M.J., Nakayama K. (1992). The Role of Attention in Different Visual-Search Tasks. Percept. Psychophys..

[13] Franconeri S.L., Simons D.J. (2003). Moving and Looming Stimuli Capture Attention. Percept. Psychophys..

[14] Wolfe J.M., Friedman-Hill S.R., Stewart M.I., O’Connell K.M. (1992). The Role of Categorization in Visual Search for Orientation. J. Exp. Psychol. Hum. Percept. Perform..

[15] Treisman A., Gormican S. (1988). Feature Analysis in Early Vision: Evidence from Search Asymmetries. Psychol. Rev..

[16] Julesz B. (1981). Textons, the Elements of Texture Perception, and Their Interactions. Nature.

[17] Treisman A.M., Gelade G. (1980). A Feature-Integration Theory of Attention. Cogn. Psychol..

[18] Wolfe J.M., Cave K.R., Franzel S.L. (1989). Guided Search: An Alternative to the Feature Integration Model for Visual Search. J. Exp. Psychol. Hum. Percept. Perform..

[19] Julesz B. (1984). A Brief Outline of the Texton Theory of Human Vision. Trends Neurosci..

[20] Fan X., Zhou Q., Xie F., Liu Z., Savage-Knepshield P., Chen J. (2017). Effects of Time Pressure and Task Difficulty on Visual Search. Proceedings of the Advances in Human Factors in Robots and Unmanned Systems.

[21] Yu R., Yang L., Guo X., Zhang Y. (2015). Effect of Time Pressure on Dynamic Visual Search Performance. Procedia Manuf..

[22] Rice S., Trafimow D. (2012). Time Pressure Heuristics Can Improve Performance Due to Increased Consistency. J. Gen. Psychol..

[23] Radović T., Rieger T., Manzey D. (2022). A Global and Local Perspective of Interruption Frequency in a Visual Search Task. Front. Psychol..

[24] McCarley J.S. (2009). Effects of Speed–Accuracy Instructions on Oculomotor Scanning and Target Recognition in a Simulated Baggage X-Ray Screening Task. Ergonomics.

[25] Van Herpen E., Trijp H.C.M.V. (2011). Front-of-Pack Nutrition Labels. Their Effect on Attention and Choices When Consumers Have Varying Goals and Time Constraints. Appetite.

[26] Rieger T., Manzey D. (2022). Understanding the Impact of Time Pressure and Automation Support in a Visual Search Task. Hum. Factors.

[27] Cambronero-Delgadillo A.J., Nachtnebel S.J., Körner C., Gilchrist I.D., Höfler M. (2024). Interruption in Visual Search: A Systematic Review. Front. Psychol..

[28] Lleras A., Rensink R.A., Enns J.T. (2005). Rapid Resumption of Interrupted Visual Search: New Insights on the Interaction Between Vision and Memory. Psychol. Sci..

[29] Lleras A., Rensink R.A., Enns J.T. (2007). Consequences of Display Changes during Interrupted Visual Search: Rapid Resumption Is Target Specific. Percept. Psychophys..

[30] Van Zoest W., Lleras A., Kingstone A., Enns J.T. (2007). In Sight, out of Mind: The Role of Eye Movements in the Rapid Resumption of Visual Search. Percept. Psychophys..

[31] Godwin H.J., Benson V., Drieghe D. (2013). Using Interrupted Visual Displays to Explore the Capacity, Time Course, and Format of Fixation Plans during Visual Search. J. Exp. Psychol. Hum. Percept. Perform..

[32] Thomas L.E., Lleras A. (2009). Inhibitory Tagging in an Interrupted Visual Search. Atten. Percept. Psychophys..

[33] Rieger T., Heilmann L., Manzey D. (2021). Visual Search Behavior and Performance in Luggage Screening: Effects of Time Pressure, Automation Aid, and Target Expectancy. Cogn. Res..

[34] Zhou Y.-B., Ruan S.-J., Zhang K., Bao Q., Liu H.-Z. (2024). Time Pressure Effects on Decision-Making in Intertemporal Loss Scenarios: An Eye-Tracking Study. Front. Psychol..

[35] Gerardin P., Nicolas J., Farnè A., Pélisson D. (2015). Increasing Attentional Load Boosts Saccadic Adaptation. Invest. Ophthalmol. Vis. Sci..

[36] Faul F., Erdfelder E., Buchner A., Lang A.-G. (2009). Statistical Power Analyses Using G*Power 3.1: Tests for Correlation and Regression Analyses. Behav. Res. Methods.

[37] Bouma H. (1970). Interaction Effects in Parafoveal Letter Recognition. Nature.

[38] The jamovi project Jamovi 2025.

[39] Cousineau D. (2005). Confidence Intervals in Within-Subject Designs: A Simpler Solution to Loftus and Masson’s Method. TQMP.

[40] Morey R.D. (2008). Confidence Intervals from Normalized Data: A Correction to Cousineau (2005). TQMP.

[41] Bahill A.T., Clark M.R., Stark L. (1975). The Main Sequence, a Tool for Studying Human Eye Movements. Math. Biosci..

[42] Lebedev S., Van Gelder P., Tsui W.H. (1996). Square-Root Relations between Main Saccadic Parameters. Invest. Ophthalmol. Vis. Sci..

[43] Pundlik S., Woods R., Luo G. (2015). From Small to Large, All Saccades Follow the Same Timeline. J. Vis..

[44] Schmidt D., Abel L.A., Dell’Osso L.F., Daroff R.B. (1979). Saccadic Velocity Characteristics: Intrinsic Variability and Fatigue. Aviat. Space Env. Med..

[45] Bollen E., Bax J., van Dijk J.G., Koning M., Bos J.E., Kramer C.G., van der Velde E.A. (1993). Variability of the Main Sequence. Invest. Ophthalmol. Vis. Sci..

[46] Guadron L., Van Opstal A.J., Goossens J. (2022). Speed-Accuracy Tradeoffs Influence the Main Sequence of Saccadic Eye Movements. Sci. Rep..

[47] Di Stasi L.L., Renner R., Staehr P., Helmert J.R., Velichkovsky B.M., Cañas J.J., Catena A., Pannasch S. (2010). Saccadic Peak Velocity Sensitivity to Variations in Mental Workload. Aviat. Space Environ. Med..

[48] Shapiro K.L., Raymond J.E., Arnell K.M. (1997). The Attentional Blink. Trends Cogn. Sci..

[49] Dux P.E., Marois R. (2009). The Attentional Blink: A Review of Data and Theory. Atten. Percept. Psychophys..

[50] Adamo S.H., Cain M.S., Mitroff S.R. (2013). Self-Induced Attentional Blink: A Cause of Errors in Multiple-Target Search. Psychol. Sci..

[51] Sigman M., Gilbert C.D. (2000). Learning to Find a Shape. Nat. Neurosci..

[52] Hu J., Chen Q., Lu D., He J. (2024). The Impact of Clock Timing on VDT Visual Search Performance under Time Constraint. Front. Psychol..

[53] Ernst D., Wolfe J.M. (2022). How Fixation Durations Are Affected by Search Difficulty Manipulations. Vis. Cogn..

[54] Von Mühlenen A., Müller H.J., Müller D. (2003). Sit-and-Wait Strategies in Dynamic Visual Search. Psychol. Sci..

[55] Wolfe J.M., Dodd M.D., Flowers J.H. (2012). When Do I Quit? The Search Termination Problem in Visual Search. The Influence of Attention, Learning, and Motivation on Visual Search.

[56] Meiran N. (2010). Task Switching: Mechanisms Underlying Rigid vs. Flexible Self-Control. Self Control in Society, Mind, and Brain.

[57] Wolfe J., Cain M., Aizenman A. (2019). Guidance and Selection History in Hybrid Foraging Visual Search. Atten. Percept. Psychophys..

[58] Liston D.B., Stone L.S. (2008). Effects of Prior Information and Reward on Oculomotor and Perceptual Choices. J. Neurosci..

[59] Zhang J., Gong X., Fougnie D., Wolfe J.M. (2017). How Humans React to Changing Rewards during Visual Foraging. Atten. Percept. Psychophys..

[60] Wagner J., Zurlo A., Rusconi E. (2024). Individual Differences in Visual Search: A Systematic Review of the Link between Visual Search Performance and Traits or Abilities. Cortex.

[61] Robson S.G., Tangen J.M., Searston R.A. (2021). The Effect of Expertise, Target Usefulness and Image Structure on Visual Search. Cogn. Res..

